# Implications of SARS-CoV-2 Infection in Systemic Juvenile Idiopathic Arthritis

**DOI:** 10.3390/ijms23084268

**Published:** 2022-04-12

**Authors:** Laura Marinela Ailioaie, Constantin Ailioaie, Gerhard Litscher

**Affiliations:** 1 Department of Medical Physics, Alexandru Ioan Cuza University, 11 Carol I Boulevard, 700506 Iasi, Romania; lauraailioaie@yahoo.com (L.M.A.); laserail_mail@yahoo.com (C.A.); 2 Research Unit of Biomedical Engineering in Anesthesia and Intensive Care Medicine, Research Unit for Complementary and Integrative Laser Medicine, Traditional Chinese Medicine (TCM) Research Center Graz, Department of Anesthesiology and Intensive Care Medicine, Medical University of Graz, Auenbruggerplatz 39, 8036 Graz, Austria

**Keywords:** autoinflammation, children, COVID-19, cytokines, CSS, damage-associated molecular patterns (DAMPs), hyperinflammation, MAS, MIS-C, pathogen-associated molecular patterns (PAMPs)

## Abstract

Systemic juvenile idiopathic arthritis (sJIA) is a serious multifactorial autoinflammatory disease with a significant mortality rate due to macrophage activation syndrome (MAS). Recent research has deepened the knowledge about the pathophysiological mechanisms of sJIA-MAS, facilitating new targeted treatments, and biological disease-modifying antirheumatic drugs (bDMARDs), which significantly changed the course of the disease and prognosis. This review highlights that children are less likely to suffer severe COVID-19 infection, but at approximately 2–4 weeks, some cases of multisystem inflammatory syndrome in children (MIS-C) have been reported, with a fulminant course. Previous established treatments for cytokine storm syndrome (CSS) have guided COVID-19 therapeutics. sJIA-MAS is different from severe cases of COVID-19, a unique immune process in which a huge release of cytokines will especially flood the lungs. In this context, MIS-C should be reinterpreted as a special MAS, and long-term protection against SARS-CoV-2 infection can only be provided by the vaccine, but we do not yet have sufficient data. COVID-19 does not appear to have a substantial impact on rheumatic and musculoskeletal diseases (RMDs) activity in children treated with bDMARDs, but the clinical features, severity and outcome in these patients under various drugs are not yet easy to predict. Multicenter randomized controlled trials are still needed to determine when and by what means immunoregulatory products should be administered to patients with sJIA-MAS with a negative corticosteroid response or contraindications, to optimize their health and safety in the COVID era.

## 1. Introduction

The current context is the crisis triggered by Coronavirus 2019 (COVID-19), set off by SARS-CoV-2, a coronavirus associated with severe acute respiratory syndrome, which has generated an uncontrolled pandemic with a significant mortality rate worldwide [1].

Management of the global health crisis has demonstrated the weaknesses and inability of the medical systems to manage this event with negative and unpredictable effects. Millions of lives have been lost, especially among the elderly, but also among children and young people, through the so-called “cytokine storm” (CS) with different clinical forms, which could not be sufficiently controlled with any pharmacological product available [2,3,4,5]. The discovery of new therapies is essential, as the global crisis triggered by the COVID-19 pandemic continues to manifest itself in various forms, even though there are currently several types of vaccines available and other potential therapeutic options [6,7]. Today’s pandemic has brought many provocations for humanity in all fields, from which pediatric rheumatology is not excluded.

Applying innovative diagnostic and therapy techniques in connection with the immune system and viral pathology is essential to meet the greatest medical challenges today, and these topics will be discussed in this review.

Complex interventions may improve the activity of the immune system and save the lives of people with immune imbalances, such as children suffering from sJIA.

The first goal of this review, in the COVID era, is to deepen the comprehension of the pathophysiological mechanisms of sJIA and to highlight the best diagnostic methods, including MAS, a life-threatening condition. The second purpose is to correctly identify all symptoms and signs of MAS in sJIA, and hyperinflammation in COVID-19, to rule out the mimickers. The last aim, but not the least, is to raise awareness among researchers, general practitioners and pediatric rheumatologists about current treatment choices and to understand the targeted approach to sJIA-MAS therapy in this developing pandemic, to provide patients with the best care to keep their immune system as balanced as possible.

In December 2019, a respiratory illness of unknown cause occurred in Wuhan, Hubei Province, China. This disease, manifested as severe pneumonia in many cases, spread to many parts of China and then outspread rapidly to many countries around the world, creating an endemic state of emergency, which soon turned into a pandemic [8,9]. On 7 January 2020, the Chinese authorities announced the identification of a new type of RNA coronavirus in broncho-alveolar lavage of sick patients, temporarily named 2019-nCoV, and on 11 February 2020, the disease was officially named COVID-19, which the World Health Organization (WHO) declared a global public health emergency in the following weeks [10]. The WHO Expert Group on Virology, Microbiology, Nomenclature and Communication recommended naming the virus SARS-CoV-2, and a nomenclature system for each new variant to appear would use the letters of the Greek alphabet [11]. Globally, from the first cases of illness to 25 February 2022, more than 430,257,564 cases of COVID-19 have been confirmed, including more than 5.92 million deaths reported to WHO. As of 20 February 2022, a total of 10,407,359,583 doses of vaccine have been administered [12].

Thus far, available data to date show that SARS-CoV-2 infections in children and adolescents are fewer and less severe than in adults, except in newborns [13]. These categories of patients may have prolonged clinical symptoms post-infection with SARS-CoV-2 (called long-COVID-19), however, current data are limited by the lack of control groups [14]. A severe post-infectious complication after SARS-CoV-2 infection that may occur in children and adolescents 3–4 weeks after the onset of a mild or asymptomatic form of COVID-19, is the multisystem inflammatory syndrome [15]. Initial studies in March 2020 showed that pediatric patients had minor symptoms, manifested by fever and cough, compared to infected adults. At the same time, asymptomatic infections were common and highlighted the high transmission potential of asymptomatic patients, which is why it was necessary to implement measures to control the ongoing pandemic. Although children did not develop major forms of the disease and few of them died, a very small number of children, even though they initially had mild symptoms or were even asymptomatic, presented later, about four weeks after a SARS-CoV-2 infection, with a severe inflammatory reaction [16].

Laboratory data for a positive diagnosis indicated that most patients, although negative for SARS-CoV-2 at the time of testing, had positive IgM or IgG antibodies, or both, that demonstrated a previous infection with this virus. The SARS-CoV-2 epidemic was associated with a 30-fold higher incidence of Kawasaki-like disease and patients had a severe progression of this, including Kawasaki disease shock syndrome (KDSS) and MAS [17]. This medical emergency was originally described in April 2020 as a disease similar to Kawasaki vasculitis (because patients had rashes, conjunctivitis, mucositis and heart damage), and was later referred to in Europe as Pediatric Multisystem Inflammatory Syndrome, temporarily associated with SARS-CoV-2 (PIMS-TS) and MIS-C in the USA [17,18,19,20,21].

Data from the literature show that MIS-C in patients infected with SARS-CoV-2 includes elements not found in Kawasaki disease (KD), such as elevated serum ferritin, cytopenia, clotting disorders, degradation of the function of internal organs, manifestations similar to CSS, MAS or secondary hemophagocytic lymphohistiocytosis (HLH), common complications in children with sJIA [22,23,24]. MIS-C was present in a patient under the age of 21, who had a high fever lasting 24 hours or more, accompanied by severe clinical symptoms that require hospitalization for multi-organ damage. Added to this are the modified biological parameters, which confirm an important inflammatory process, with evidence of recent contamination with SARS-CoV-2, or a present infection confirmed as COVID-19, within 1 month before the onset of symptoms. Clinical manifestations are severe, expressed by cardio-respiratory and renal insufficiency, to which is added intravascular coagulation, coronary artery dilation, aneurysms, etc. Unlike KD, patients with extremely severe forms of MIS-C experience vasodilator or cardiogenic shock that requires cardio-respiratory resuscitation, mechanical ventilation and extracorporeal membrane oxygenation. Biological data show an increase in: C-reactive protein (CRP), the erythrocyte sedimentation rate (ESR), fibrinogen (Fg), D-dimers, serum ferritin, procalcitonin, lactic acid dehydrogenase (LDH), neutrophils, interleukin 6 (IL-6), with lymphopenia and hypoalbuminemia, and changes that are not necessarily present for all parameters [25]. Because there are significant differences between these two syndromes, it was disputed whether to consider MIS-C a new disease, or as a “severe type” of KD [26].

The American College of Rheumatology (ACR) expert group has approved the Clinical Guidance for Multisystem Inflammatory Syndrome in Children Associated with SARS-CoV-2 and Hyperinflammation in Pediatric COVID-19, Version 1 and 2, as a “living document” stipulating the management of MIS-C, but also the recommendations for hyperinflammation during the acute, infectious phase of SARS-CoV-2 infection [27,28].

MIS-C was considered to be caused by a hyperinflammatory response to infection in some children. Even if there is no specific diagnostic test, the condition is accompanied by persistent fever and increased values for the biological parameters of inflammation. Although MIS-C is unusual, with the estimated incidence to be 11.4 per 100,000 persons younger than 21 years from 1 March to 30 June 2020 [29], many children around the world have suffered from the disease to date.

The innate immune system initiates an excessive and uncontrolled inflammatory response, triggering a cytokine storm [30,31].

The study and accumulation of data for this new syndrome, which proves fatal in 1.8% of cases [20], was rapid, and although initially the clinical features appeared like KD, it has been shown that there are important differences between these two disorders [32,33].

## 2. Systemic Juvenile Idiopathic Arthritis

Systemic juvenile idiopathic arthritis is a subtype of childhood-onset juvenile arthritis that has high morbidity and serious complications, especially in patients who do not respond to treatment. This highly inflammatory disease is manifested by high, prolonged fever, unaffected by antibiotics, which is associated with rashes in febrile acme, sometimes progressing to devastating chronic polyarthritis [34,35].

Incidence of JIA varies from 1.6 to 23, and the prevalence is from 3.8 to 400 cases/100,000 children/year, of which the prevalence of JIA is estimated between 0–8.6 cases/100,000 children. These statistics vary from country to country, as reported by the authors [36,37,38,39].

JIA with systemic manifestations that affect both girls and boys alike, formerly known as Still’s disease, accounts for 4–20% of all cases of juvenile idiopathic arthritis [35,40]. Moreover, sJIA is a multifactorial autoinflammatory disease with characteristic clinical symptoms that differs from other subtypes of JIA by the presence of prominent systemic inflammatory phenomena, which may include arthritis.

Compared to the classification criteria for systemic arthritis with onset in adulthood, i.e., adult-onset Still’s disease (AOSD), Martini et al. proposed among the new classification criteria for sJIA, that arthritis should no longer be necessary as benchmark, in the context that systemic inflammation is the defining feature of this subtype of the disease [35].

sJIA is manifested by autoinflammatory rather than autoimmune symptoms: prolonged fever for more than two weeks, evanescent rash (unfixed), lymphadenopathy, hepato-splenomegaly, serositis, arthralgia and/or arthritis (Table 1), similar to the onset of AOSD, but in young adulthood [37,41].

Diagnosis of this subtype of arthritis is established based on two broad categories of clinical and biological criteria: major and minor. Major criteria include evanescent rash and arthritis. Minor criteria consist of generalized adenomegaly and/or splenomegaly and/or hepatomegaly, arthralgia lasting at least two weeks in the absence of arthritis, serositis (pleural, pericardial or peritoneal) and leukocytosis (≥15,000/mm^3^) with neutrophilia. Proposed criteria for diagnosis take into account the cardinal symptom of a prolonged fever of unknown origin (only when infectious, neoplastic, autoimmune or monogenic diseases or other autoinflammatory diseases are excluded). Fever of unknown origin, prolonged, daily, in bursts of ≥39 °C with a return to ≤37 °C, for at least three consecutive days, which is repeated for a period of at least two weeks, in which two major criteria can be proved or one major criterion and two minor criteria, will signify the diagnosis of sJIA (Table 2).

The criteria for a positive diagnosis and classification of JIA subtypes, according to the International League against Rheumatism, and for sJIA, are those given by the clinical manifestations of the first six months of the disease with the presence of arthralgia or arthritis. In sJIA, in addition to a prolonged fever, at least one major criterion is absolutely necessary; the presence of arthritis and for the duration of six weeks that were previously mandatory, after a new consensus, are no longer necessary because some patients do not have arthritis but have classic systemic manifestations of sJIA. At the same time, for a similar disease in adults, according to the Yamaguchi criteria, only the presence of arthralgias for more than two weeks associated with systemic symptoms is sufficient to confirm a positive diagnosis [34,35,42,43,44].

Since we do not have a biological test available to diagnose sJIA or AOSD, the clinician must diagnose by excluding other diseases with similar symptoms, based on the anamnesis data, clinical signs and the following biological parameters: blood count, increased ESR and CRP, elevated serum ferritin levels, elevated levels of interleukin 1 (IL-1) and IL-6. Rheumatoid factor is usually negative in sJIA and may be negative in AOSD [34,35,42,45].

A positive diagnosis involves the exclusion of other diseases with similar symptoms, such as the following: infections, malignancies, autoimmune diseases, vasculitis, immunodeficiencies, recurrent febrile autoinflammatory diseases [46,47,48,49]. An interesting aspect in establishing a positive diagnosis is the therapeutic test by administering antibiotics, as a result of which no response is obtained; while after corticosteroids administration, the fever is cut off immediately—which is a valuable sign for the diagnosis of sJIA [50,51]. Imaging techniques (ultrasonography, X-ray radiography, computed tomography, nuclear magnetic resonance imaging, thermography, scintigraphy, etc.) are very useful, both in establishing a positive diagnosis and for monitoring the treatment and evolution of the disease [52].

Exclusion criteria in the differential diagnosis of juvenile arthritis subtypes encompasses: the presence of the IgM-type rheumatoid factor in at least two determinations over a 3-month interval; psoriasis or a history of psoriasis in the patient or a family history; arthritis manifested in a male patient with a positive HLA-B27; symptoms of ankylosing spondylitis, arthritis-related enthesitis, sacroiliitis with inflammatory colopathy, reactive arthritis (Fiessinger-Leroy-Reiter syndrome) with acute anterior iridocyclitis or a positive, if not family or personal history [53,54].

The etiologic factor determining the onset of sJIA in children as well as adult-onset Still’s disease is unknown, but there is evidence of several mechanisms associating genetic predisposition and infectious agents, which trigger and maintain a hard-to-reduce inflammatory process [55]. The role of genes encoding the human leukocyte antigen (HLA) is located in the major histocompatibility complex (MHC) region, most of which are essential to the immune system. The genes printing a genetic predisposition in juvenile arthritis have been clearly documented, but some studies show that they do not exceed 18% [56,57]. The list of coding genes that do not relate to the HLA system includes about 200 names, but the confirmed connections have only been certified for single genes [56,58]. Tadaki et al. genetically investigated disease-related copy number variations (CNVs) by single nucleotide polymorphism analysis in 50 patients with sJIA. Most CNVs found were inherited from either parent with a normal phenotype; only one patient identified two de novo microduplications at 19q13.42, which included the NLRP (Nucleotide-binding oligomerization domain, Leucine-rich Repeat and Pyrin domain) containing family (NLRP2, NLRP9 and NLRP11), as well as IL-11 and HSPBP1 gene (Hsp70-binding protein 1), all of which play a major role in inflammatory pathways and sJIA pathogenesis. Although the MIF (macrophage migration inhibitory factor), IL-6, IL-10 and TNF genes have been associated with sJIA in different populations and subtypes, these genes are still a small part of the total genetic range contributing to the etiopathogenesis of sJIA [59]. Today, mutations in the genes that cause activation of the inflammasome or interleukin pathways, along with NOD-like receptor pyrin domain-containing protein 3 (NLRP3), TNF cytokine receptor 1 (TNFR1), and IL-1 receptor antagonist are major links in the etiopathogenesis of autoinflammatory diseases, including sJIA and AOSD [60].

Pathogenesis of sJIA has, as its fundamental primary support, the involvement of the innate immune system, to which is added the association of TNF, IL-6, IL-10, MIF, IL-1, and predominant effector cells, such as monocytes, macrophages and neutrophils. Primum movens in the pathophysiology of sJIA would be the abnormal activation of phagocytes that cause a cascade of hypersecretion of proinflammatory cytokines. Proinflammatory cytokines include IL-1, IL-6, IL-18, IL-37, leucine-rich α2-glycoprotein 1 (LRG1) and adenosine deaminase-2 (ADA2). Data from the current literature demonstrate through more and more studies the autoinflammatory mechanisms in sJIA pathogenesis, with a disruption of the innate immune system, stimulation of excessive activation of macrophages, neutrophils and hypersecretion of cytokines and proinflammatory proteins (S100A8, S100A9 and S100A12). MIF gene encrypts a lymphokine involved in innate immunity, immune-adjustment and inflammation by establishing the function of macrophages. Pro-inflammatory active, it is involved in the innate immune response with an important role as a mediator in regulating the activity of macrophages in the defense of the host. MIF serum and synovial fluid levels are higher in arthritis [61,62,63,64,65,66].

Through the innate immune system, natural killer (NK) cells have the crucial role of defending the body through their power to destroy foreign cells that are infected, but also malignant ones, by forming a lytic immune synapse with the target cell and releasing some strongly cytotoxic molecules (perforin and granzyme B) [64,67].

Another ability of NK cells is the immunomodulation and regulation of cytokine release, mainly interferon-gamma (IFN-γ) and TNF-α, as well as the removal of activated and autoreactive autologous immune cells in order to stop an inflammatory process [64,68].

NK cells’ activity is implicitly related to the existence of a balance between activator and inhibitor receptors expressed on their surface, the activation of cytokines IL-2, IL-12, IL-15, IL-18 and the expression of the corresponding ligands of the target cells.

Subtle NK cell inhibitory and activating receptors defects have been observed in patients with sJIA, with decreased KLRG1 (killer cell lectin-like receptor subfamily G member 1) inhibitory receptor expression and increased NKp44 (gene: NCR2, synonym CD336) activator receptor expression [69].

IL-18, together with IL-12, activate CD4+T, CD8+T and NK cells to induce the production of IFN-γ, a type II interferon which in turn has a special role in activating macrophages or other cells. It is assumed that, in sJIA, IL-18 participates in the increase of IFN-γ, with the consequence of dysfunction and depletion of NK cells and the appearance of MAS. Thus, a disorder of NK cell function in sJIA is one of the most important causes of association with macrophage activation syndrome [70,71]. To date, the role of these cells, molecules, complex activation pathways and pathophysiological mechanisms in sJIA are not yet well known.

Other causes involved in the pathogenesis of sJIA include the quantitative reduced and also defective secretion of IL-10 by B lymphocytes, demonstrated in experimental studies in animals with chronic inflammation and gamma interferon deficiency [72].

The activity of IL-10 is known for its strong anti-inflammatory properties, which effectively participate in stopping the host’s immune response to pathogens, protecting both the host and the normal balance of tissues. Disruption of IL-10 function is associated with a high risk for a wide variety of autoimmune diseases [73].

IL-10 is a pleiotropic cytokine released by a multitude of cells being able to stop the synthesis of IFN-γ, IL-2, IL-3, TNF-α and GM-CSF, proinflammatory cytokines produced by macrophages and Th1 cells. At the same time, it works for the survival and proliferation of B cells, and antibodies generators; however, may inhibit the activity of the NF-κB inflammatory pathway and participates in the regulation of the JAK-STAT signaling pathway [74,75].

During the proinflammatory period of macrophage activation, rapid transcription of mRNA by IL-10 was observed, and the inhibitory action of IL-10 resulted in a strong deprivation of immunoregulatory markers, with a concomitant increase in proinflammatory surface markers [76,77].

An interesting aspect of the evolution of the disease is that, unlike other autoinflammatory diseases, sJIA is often self-limited because between 30% and 50% of patients have a form of “single-phase sJIA”, i.e., a single episode, which ends in 24 months; the clinical expression of this phenotype may prove biologically the restoration of immune tolerance. In general, sJIA evolves in stages, with the appearance of “saw teeth”, with characteristic systemic symptoms, such as fever, skin evanescent rash, arthritis, manifestations that disappear and reappear over time, and thus defining a course of polycyclic disease (10%), single-phase with variable duration (40%) and persistent (50%) [78], in the latter case leaving behind a chronic disease sometimes manifested by intractable arthritis [79].

Gurion et al. have published a review that looked at the epidemiology, pathogenesis, genetics, clinical manifestations, complications, therapy, prognosis and outcome in sJIA. This review demonstrates that the evolution and prognosis of sJIA vary considerably from the monocyclic mode with a good evolution to a more severe one, which attracts considerable morbidity or mortality. It has been observed that 50% of patients with sJIA have a monocyclic evolution, in which the physical damage is minimal, and the recovery is achieved in 2–4 years [80].

Reduction in systemic and joint symptoms has been observed in some patients with recurrent sJIA, who over time may have a complete resolution of systemic manifestations, but remain with persistent arthritis, which may improve within 5 years of onset [81,82].

Overall, considering all cases of sJIA, it was observed that approximately 30% have a course manifested over time by polyarticular damage to the small joints of the hands and feet bilaterally, but also to large joints and the spine, especially the cervical area, and temporomandibular with intensely destructive characteristics, responsible for morbidity and very poor prognosis [83,84].

Usually, this category of patients shows more severe clinical signs for more than 10 years from the onset of the disease and continues into adulthood [85].

Elements with a poor prognosis include a low age at onset (around 6 months), generalized adenomegaly, thrombocytosis and early involvement of the cervical spine, small joints and hip [86,87,88,89].

Another formidable complication remains MAS, in which deaths are between 8–22% of cases [90,91].

Stoeber studied 433 children with chronic arthritis, admitted to the Rheumatic Children’s Hospital Garmisch-Partenkirchen, which he monitored for an average of 15 years (range 10–22 years) after a positive diagnosis; of the total number of patients studied, 13.4% had Still’s syndrome. It should be noted, that the mortality in the systemic group was 13.8%, in contrast to 1% in the non-systemic polyarticular group, and 0% in those with minor joint forms. In systemic cases, secondary amyloidosis was the leading cause of death; of the patients with amyloidosis, 44% died in the second or third decade of life due to chronic renal failure with terminal uremia [92].

If a few decades ago secondary or generalized renal amyloidosis was a formidable cause of death for patients with sJIA, this risk has greatly diminished or even disappeared in some countries [81,93].

Additionally, other complications that should not be overlooked are: psychological (anxiety, fear, feelings of revolt, depression, etc.), social (isolation, poor academic competition, dropping out of school, etc.), quality of life and economic (limitation of professional specialization, integration into work, impoverishment, etc.) [94,95].

The vital prognosis of this subtype of arthritis depends on the severity of systemic manifestations, especially MAS, disseminated intravascular coagulation, and lung damage due to acute respiratory distress syndrome with multiple organ failure which is caused mainly by SARS-CoV-2 infection. Physicians should take great care of children with sJIA and be aware of the occurrence of a hyperinflammatory syndrome associated with COVID-19 in this category of patients, as this pathology may be easily confused with the primary disease and may develop severely [81,92,96].

Barut et al. aimed to retrospectively investigate the demographic and clinical characteristics, the response to long-term treatment and the complications of a group of 168 patients with sJIA for a period of 15 years (2003–2017). Results showed the evolution of monocyclic type in 31.5% of cases, 13.7% polycyclic and 54.8% persistent cases. All patients received corticosteroids at the beginning of the disease, methotrexate in 75% of cases, cyclosporine A in 17.3% cases, and biologics in 42.8% of patients (29.7% etanercept, 16% anakinra, 16% canakinumab, and 10% tocilizumab). MAS was manifested in 11.9% of cases. Among the complications that could be mentioned: 11.3% of cases delayed growth, 8% had osteopenia and three patients (1.78%) died due to MAS secondary multi-organ insufficiency and infection. Authors concluded that steroid therapy is the basic pillar at the onset of the disease; biologics should be administered in a timely manner, and MAS was the major mortality factor [97].

Schulert et al. investigated the clinical symptoms, risk factors, and histopathological and immunological features of lung disease (LD) associated with sJIA. For a period of 9 years, 18 patients with sJIA-LD were identified from the records of Cincinnati Children’s Hospital Medical Center. The radiological examination was characterized by diffuse ground-glass opacities, subpleural reticulation, a thickening of the interlobular septum and lymphadenopathy; there were also detected irregular lymphoplasmacytic infiltrates, combined characteristics of pulmonary alveolar proteinosis (PAP) and endogenous lipoid pneumonia (ELP). Additionally, sJIA-LD patients had a low age upon their confirmation of sJIA diagnosis, more episodes of MAS, higher serum IL-18 concentrations (median 27,612 pg/mL versus 5413 pg/mL), and a higher frequency of adverse reactions to biological therapy, compared to patients without sJIA-LD. Broncho-alveolar lavage (BAL) fluid in patients with sJIA-LD had elevated levels of IL-18 and IFN-γ-induced chemokines, CXCL9 and CXCL10; increased T cell and IFN type II activated networks were identified in lung tissue. The authors concluded that LD is increasingly being detected recently in patients with sJIA, especially in those with MAS [98].

In another multicenter study, Saper et al. retrospectively investigated 61 cases for characteristic symptoms and risk factors of a new parenchymal LD, more and more frequently detected in the current evolution of sJIA. The authors found specific features by increased serum ferritin and/or lymphopenia, acute urticarial skin lesions and/or anaphylactic-type reactions, and characteristic thoracic CT lesions (thickening of the alveolar septa, ground-glass opacities, etc.) secondary to the treatment with tocilizumab (TCZ), an IL-6 inhibitor. Of the patients undergoing IL-1 and IL-6 inhibitors, 46 of the 61 cases had multiple features of LD, suggesting macrophage dysfunction. Furthermore, 23 out of 36 cases had PAP and/or ELP, with atypical images of regional damage and associated vascular changes. Survival at 5 years was 42% of cases [99].

## 3. Macrophage Activation Syndrome in sJIA

Current research has already shown that there is a strong activation of the innate immune system in the onset of symptoms in sJIA, and the proinflammatory cytokines IL-1 and IL-6 actually participate in multisystemic inflammation and may contribute to MAS, a severe complication found in 10% of patients [100].

MAS is considered a secondary form of HLH, which can be triggered in over 20% of cases at the onset of sJIA after a change in treatment, through infection, or even spontaneously; it can be occult or subclinical in another 30–40% of cases. From a clinical-biological point of view, MAS is expressed by: non-remissive high fever at classical medication, pancytopenia, splenomegaly, hepatomegaly, significant hepatic cytolysis, coagulopathy, the presence of hemophagocytic macrophages in the bone marrow, hyperferritinemia, disruption of the activity of the central nervous system and the disturbance of innate immunity through the decrease of NK cell cytolytic function [101,102,103,104].

Raveli et al. [105] issued criteria for classifying MAS in patients with sJIA based on a general consensus of a group of 28 EULAR, ACR and PRINTO experts and by analyzing actual patient data. During the 2016 conference, a consensus was reached on the final MAS classification criteria, which are still valid today (Table 3).

The fact that the innate immune system is strongly activated, and it releases an increased amount of IL-1 and IL-6 participating in multisystemic inflammation and the onset of MAS, has led to the administration of IL-1 blockers in the treatment of sJIA (e.g., Anakinra or canakinumab) and IL-6 inhibitors (TCZ), and giving up TNF-α inhibitory biologics, which is further evidence of an autoinflammatory process [106,107].

Although the precise mechanisms involved in the onset of MAS are not well known, there is evidence to support the role of IL-18 in IFN-γ release, and elevated IL-18 levels may explain the link between hyperactivity of the inflammasome and secondary HLH/MAS. Thus, IFN-γ-induced biomarkers such as the chemokines CXCL10 or CXCL9 can be considered valuable biomarkers in the test range for the diagnosis of sJIA and secondary MAS [108,109,110].

Guo et al. conducted a retrospective study of 149 patients with sJIA, where 27 of whom had 31 episodes of MAS to find predictive clinical-biological parameters for early MAS. A high percentage of patients with fever and dysfunction of the central nervous system was detected in the absence of arthritis at the onset of MAS; at the same time, 35% of MAS patients had hypotension without hypovolemic shock, and 22.6% of MAS patients had gastrointestinal involvement at the onset of MAS. At the same time, platelet counts, serum LDH and AST levels helped to identify early MAS, and the ESR/serum ferritin ratio of approximately 20.00 had a high sensitivity and diagnostic specificity for MAS. In addition, the authors noted that the level of IFN-γ > 17.1 pg/mL and IL-10 > 7.8 pg/mL appears to be a good model of cytokines for recognizing the onset of MAS. In conclusion, sudden hypotension, high ESR/serum ferritin ratio, and significantly elevated levels of IFN-γ and IL-10 are valuable markers for the early diagnosis of MAS [111].

Disruption of NK cell function has been considered a common pathology for the onset of sJIA, MAS, and also for HLH, which is very similar to MAS. To date, the molecular basis of immune dysfunction and the etiology of sJIA and MAS remains unclear, as genetic and genomic investigations are restricted by small samples of patients undergoing the study [112,113,114].

Zhou et al. used the weighted gene co-expression network analysis (WGCNA) to identify co-expression modules related to sJIA and non-sJIA patients and studied the hub genes in the modules. The results of the study show that the authors identified key genes associated with sJIA, such as ALAS2, AHSP, TRIM10, TRIM58, and KLF1, which are largely related to red blood cell differentiation. These genes may be linked to sJIA and MAS anemia. Moreover, the identified KLRB1, KLRF1, CD160 and KIR genes would be correlated with NK cell dysfunction. The authors believe that identified hub genes may serve as biomarkers and therapeutic targets for sJIA in the future [115].

An important factor in triggering MAS is thought to be a defect in the cytolytic capacity of lymphocytes. Thus, it was found that some mutations in the cytolytic pathway genes, such as perforin 1 (PRF1) and Unc-13 Homolog D (UNC13D) in the HLH family, were linked to MAS and the presence of proinflammatory interleukins, especially IL-6, which depresses cytolytic capacity of the NK cells. These functional disturbances of NK cells and cytolytic CD8+T lymphocytes, to destroy the infected and activated antigen-presenting cells (example: monocytes/macrophages), will lead to a prolonged process of intercellular interactions with exacerbation of the proinflammatory cytokine cascade [104,116,117].

## 4. MAS in sJIA and Hyperinflammation in COVID-19

From the published data so far, which is related to MAS and the cytokine storm from a viral infection with SARS-CoV-2 and other viral infections, for example, H1N1, there are multiple common aspects of biochemical and immunological abnormalities. Biological parameters show disorders in blood clotting with increased D-dimers and serum ferritin, high von Willebrand factor, intravascular coagulation with microthrombus formation, and increased vascular endothelial permeability [118,119,120].

MAS, a severe complication of sJIA, seems to overlap with CSS and has appeared since 2020 in a certain category of patients with COVID-19. The term CSS brings together clinical and biological manifestations at the same time as hyperinflammation, which can compromise the hemodynamic activity and functions of multiple organs, with disastrous consequences for the patient. In several published studies, the authors consider MAS as a prototypical form of CSS that appears as a severe complication in the evolution of several autoinflammatory and/or autoimmune diseases, such as sJIA, AOSD, SLE and KD [121,122,123].

Analyzing MAS in patients with sJIA, it was found that from a clinical-biological point of view, these data are accompanied by an exaggerated hyperferritinemia, coexisting with hemocytopenia, and coagulopathy of consumption with significant hepatic dysfunction. The predictive prototype for MAS in the febrile patient with sJIA is hyperferritinemia (>684 ng/mL), which will be associated with any two of the following additional criteria: platelet count ≥181 × 10^−9^/L, aspartate aminotransferase >48 units/L, triglycerides >156 mg/dL and fibrinogen ≥360 mg/dL. Since hyperinflammation in sJIA is of particular importance and the practitioner must assess in a timely manner whether it is intensely active sJIA or whether MAS is already occurring, differentiation guidelines have been implemented to support the clinician [105,124,125].

The current prototype supporting MAS pathogenesis is based on the involvement of IL-18 with the overproduction of IFN-γ and the inability of NK cells and cytolytic CD8+ T cells to lyse infected and activated antigen-presenting cells. Prolonged interaction between innate and adaptive immune cells will amplify a cascade of proinflammatory cytokines, which will stimulate macrophages in the process of hemophagocytosis and multisystem dysfunction [104]. It has been observed that in MAS, the serum concentration of IL-18 is decisive for the amplification and perpetuation of the degree of activation of the cells of the innate immune system, and its plasma level can differentiate between sJIA complicated by MAS, and other inherited (monogenic) periodic fever syndromes [126]. As already reported, IL-18 is known to be monitored and regulated by its high-affinity natural antagonist IL-18BP, which can block its biological activity. Although both entities are under the influence of inflammatory cytokines in sJIA and MAS, as well as in viral infections or various HLH-associated diseases, a major imbalance was detected with abnormal growth of bioactive IL-18, which appears to induce IFN-γ synthesis and, consequently, the MAS emergence [127,128]. Lack of proportion in IL-18/IL-18BP concentrations gives rise to high systemic free bioactive IL-18, putting at risk MAS progress. The use of recombinant IL-18BP in patients with AOSD and sJIA with MAS has had encouraging effects for the application as useful biomarkers of IL-18, especially free IL-18, but also as attractive new medicines. Recent findings have shown that elevated levels of free IL-18 are present in correlation with clinical and biological signs of disease activity. A very interesting aspect is that some patients with these diseases have responded very well to treatment with recombinant human IL-18BP, which demonstrates the pathogenic role of Il-18 and recommends the use of IL-18 inhibitors in difficult-to-treat autoinflammatory diseases. In spite of the fact that the whole picture of MAS evolution is not fully comprehensible, the counterbalancing of level IL-18/IFN-γ in the management of severe HLH and MAS stopping could be a very good solution [129,130].

Quite surprisingly, in the first wave of SARS-CoV-2 infection in China, the clinical parameters were identical to those observed in CSS. Despite the fact that CSS has been known for over a century, it is only in the last three decades that the scientific medical world has begun to deepen and unravel the clinical and biological aspects, its name covering as an umbrella HLH, the hemophagocytic syndrome associated with infection, the cytokine release syndrome, cytokine storm and MAS. During the COVID-19 pandemic, the appearance of cases with a brusque and fast evolution towards exitus, motivated the amplification of studies on pathogenesis, entity recognition and the adequate therapy of lethal CSS [131].

Compared to SARS-CoV, which is thought to induce inadequate interferon (IFN) responses, SARS-CoV-2 robustly triggered the expression of numerous IFN-stimulated genes involved in inflammation [132].

If we make a comparison between MAS and the occurrence of COVID-19 CSS, we can say that when the human body faces a viral infection, the virus-specific cytotoxic T lymphocytes (CTLs) get involved, working together with antigen-presenting cells (APC) and macrophages for stopping and removing viral agents. Physiologically, when the process is well completed, all the activated immune cells are removed by the cytolytic cells and the immune system, so that everything returns to the initial stage of prevention. In the process of activating macrophages, the innate immune defense system in teamwork with IL-18 trains APC and the macrophages to fight. If the process of activating innate immune cells and inadequate cytolytic cells persists, then the potency of removing the activated immune cells decreases, leading to long-lasting action of CTL-APC, and the permanent expulsion of inflammatory mediators, disruption of CTL-macrophage activity and the occurrence of such a cytokine storm as MAS. In SARS-CoV-2 infection, the same antiviral immune pathways will be activated with the extended participation of CTL. Participation of type I interferon will allow the persistence of lung infection, and the activation of type II interferon will facilitate the persistent process of immune activation. If the virus cannot be eliminated, there will be a continuous signaling from type I interferon, which will result in the production of proinflammatory cytokines. Similar to in MAS, the cytolytic capacity of CLT will be disrupted due to immune depletion and/or inappropriate antiviral replication, which would maintain a prolonged disagreement between CTL, APC and the alveolar macrophages, facilitating COVID-CS [133].

It has been shown that both in MAS and HLH, but especially in severe COVID-19, it is implicated the dysfunction of type I interferon, NK cells, with extensive CTL involvement, increased IL-6, TNF-α, and excessive inflammation caused by the NF-kB pathway. Despite this similarity between the three prototypes of pathologies, there are distinct clinical features not found in MAS. According to several recent studies, severe COVID-19 is characterized in particular by clinical and biological signs of dramatic coagulopathy with complement activation, vascular endothelial lesions and microthrombosis, a pathology similar to thrombotic microangiopathy [134,135,136].

Type I interferons have a very important role in innate immunity because toll-like receptors 3, 7 and 9 (TLR3, 7 and 9) are expressed [137,138] as pattern recognition receptors (PRRs) on the surface of antigen-presenting cells (macrophages, dendritic cells) and B lymphocytes, that once activated, will express IFN, stimulate signaling pathways involved in NF-kB activation and interferon regulatory factors (IRF) IRF3 and IRF7, which will create the possibility of transcription and production of IFN-α and other proinflammatory cytokines [139].

PRRs recognize both PAMPs and DAMPs derived from tissue damage, while type I IFNs (IFN-α and IFN-β) released from the cell establish an autocrine and paracrine loop with other molecules for the antiviral defense of the infected cell. If viral agents have not been removed, then the proinflammatory state intensifies, the cellular functions are disrupted, recruitment is intensified, and the activation of adaptive effector cells and release of a massive amount of proinflammatory factors take place.

Disruption of type I interferon activity by genetic mutations or by the involvement of some autoantibodies results in failure to stop the spread of primary SARS-CoV-2 infection in the lungs, especially the invasion of the alveoli by macrophages, which, when activated, maintain the immune processes [140,141].

In patients with severe COVID-19, a strong activation of alveolar macrophages and very high pulmonary concentrations of proinflammatory mediators (IL-6, IL-8, IL-1β) and chemokines were found. In the early stage of SARS-CoV-2 infection, IFNs strongly participate in the antiviral activity by training NK cells and antigen-presenting dendritic cells, activating and differentiating virus-specific T cells, attracting memory T cells, T and B lymphocytes to the site of the impact. IFNs work antivirally by activating CTLs, inhibiting viral mRNA translation, disintegrating the viral RNA, and adjusting the nitric oxide release. Prolonged involvement of interferon to eliminate the virus facilitates the development of a self-sustaining outbreak in the lungs, which can be exacerbated by a feedback loop and will lead to a cytokine storm. Evidence of the involvement of IFNs in SARS-CoV-2 infection and recent advances in understanding the mechanisms of action have led to the development of targeted antiviral therapy with IFN-α and IFN-β. Research shows that the use of IFNs in the treatment of the early stages of the disease, more precisely before the onset of severe signs of inflammation, could be of great protective benefit to patients with COVID-19 [142,143,144].

Adachi et al. reviewed the publications using PubMed and Google Scholar for citations published after the outbreak of COVID-19, from December 2019 to July 2021, and identified six cases of JIA with COVID-19 during treatment with biologic DMARDs (anti-TNF-α or anti-IL-1β). Of the studied cases, only one patient with sJIA receiving oral prednisolone in combination with canakinumab required oxygen therapy for COVID pneumonia. The authors also presented a case of sJIA with MAS in remission for 5 years from the initiation of TCZ therapy at a dose of 8 mg/kg every 4 weeks and MTX (7 mg/m^2^) weekly, personally studied. The patient developed a fever for two days and was diagnosed by way of a positive RT-PCR test with the SARS-CoV-2 α-variant, as was her father. At hospital admission, there were no signs of respiratory failure, chest X-ray abnormalities, or altered biological parameters, except for the cytokines TNF-α, IL-6 and IL-8 being slightly increased. The patient continued to receive oral MTX without any antiviral drugs or oxygen therapy, and TCZ was reintroduced after the fever disappeared. The case presented by the authors is the first with SARS-CoV-2 infection with the α-variant under treatment with TCZ. At discharge, the disease was controlled, and from a biological point of view, only IL-6 was detected at a low level. In the end, the authors mentioned that the COVID-19 disease was cured without serious complications in all forms of JIA under biologics, but not with long steroid therapy. These data support the hypothesis that patients with active JIA under steroid immunosuppressive therapy have a risk of developing severe COVID-19 [145,146,147].

Data from the literature converges to the idea that low-dose steroid immunosuppressive therapy in patients with JIA under biologic therapy does not significantly increase the risk of severe COVID-19. On the other hand, pediatric rheumatology studies indicate that MIS-C associated with severe COVID-19 has pathophysiological similarities to hyperinflammatory abnormalities in MAS in patients with sJIA treated with TCZ. At the same time, TCZ used in patients with sJIA may be useful in severe COVID-19 pneumonia [148].

In a recent study, Aydın et al. investigated 13 patients with sJIA and MAS and 26 with MIS-C. The authors observed that patients with MAS had lower hemoglobin (10.10 g/dL) and fibrinogen (2.72 g/dL), but higher ferritin (17,863 mg/dL) and LDH (890.61 U/L) at diagnosis. Absolutely higher neutrophil counts (12,180/mm^3^) and CRP values (194.23 mg/dL) were found in patients with MIS-C, while absolute lower lymphocyte count (1140/mm^3^) were found at the time of diagnosis. The left ventricular ejection fraction was reduced in the MIS-C group. In conclusion, the authors showed that the levels of ferritin, hemoglobin, LDH and fibrinogen were significantly increased in MAS compared to MIS-C. However, patients with MIS-C had more severe signs of heart damage than those with MAS [149].

## 5. Interrelationship between SARS-CoV-2 Infection and sJIA

Effects of the ongoing coronavirus pandemic caused by severe acute respiratory syndrome coronavirus 2 (SARS-CoV-2), with symptoms ranging from none to life-threatening, have also dramatically affected the rheumatic care, the initial presentation and the research on sJIA worldwide.

Dushnicky et al. collected data (the time from the onset of symptoms to the first assessment, the severity of disease on presentation and the recruitment record) from the archive of the Canadian Alliance of Pediatric Rheumatology Investigators (CAPRI) for JIA in the pre-pandemic year (11 March 2019–10 March 2020) and compared them with data from the first year of the pandemic (11 March 2020–10 March 2021), through appropriate statistical processing. Although the mean times from the onset of disease to the first presentation were comparable, and the frequency of different categories of JIA remained stable, sJIA was an exception (12 pre-pandemic cases, and only 1 in the pandemic). Otherwise, the clinical features, disease activity, disability scores and quality of life were similar. Authors concluded that in Canada, the COVID-19 pandemic did not affect the presentation to pediatric rheumatologists of new patients with JIA due to the pre-existing telemedicine infrastructure, which prioritized virtual over in-person consultations, adapted to new circumstances [150].

The main objective of pediatric rheumatologists was to prevent the exacerbation of systemic autoinflammatory and/or autoimmune diseases in patients on chronic immunosuppressive therapies at risk of contracting, (and possibly) a more severe course of SARS-CoV-2 infection. All research has pointed out that to prevent a severe course of infection in rheumatic patients, vaccination should be widely recommended, being safe and effective [151].

The imminent danger of very severe COVID-19 has been shown to vary with age, sex and the presence of comorbidities, so that it increases exponentially with age; with a two-fold increase in a single comorbidity, or five-fold greater threat in multimorbidity (three or more comorbidities) [152].

Nearly all children with COVID-19 had mild symptoms and favorable evolution, except for MIS-C, a life-threatening illness and long-term sequelae [28,153,154].

Walters et al. investigated the seroprevalence of SARS-CoV-2 IgG and the clinical course in a big group of pediatric patients with rheumatic diseases. The majority of positive subjects had no symptoms and the symptomatic ones all had mild COVID-19 symptoms, suggesting the lowest risk of severe or critical COVID-19 in immunocompromised rheumatic pediatric patients [155].

To better guide clinicians when experiencing SARS-CoV-2 infection in pediatric rheumatic patients under immunomodulatory drugs or underlying diseases, Sozeri et al. investigated in a multicenter retrospective design, the clinical manifestations and the evolution of COVID-19 in 113 children receiving biologic disease-modifying antirheumatic drugs (DMARDs) due to their major rheumatic disorders. They were aged 12.87 ± 4.69 years and diagnosed as follows: 63 patients with JIA, 35 with systemic autoinflammatory diseases, 10 with vasculitis, and 5 cases of connective tissue disease. The mean duration of primary disease was 4.62 ± 3.65 years. Fever and dyspnea were considered risk factors for hospitalization, occurring in 21.2% of cases with a younger age, a shorter duration of the disease, and a higher rate of steroid application compared to outpatients (78.8%). The link between comorbidities (cardiovascular, hepatic, renal or malignant diseases) and a severe course of SARS-CoV-2 infection has been explored, however, is still limited for patients using biologic DMARDs. The authors concluded that there was no exacerbation of both COVID-19 and rheumatic disease under biologic DMARDs, but multicenter international studies are still needed to highlight all the risk factors for a severe course of COVID-19 among pediatric rheumatic patients [156].

As the most common chronic rheumatic disease in children, JIA has raised the question of whether these patients are at higher risk and whether their treatment should be changed for COVID-19 infection.

Boyarchuk et al. investigated the frequency of COVID-19 infection in JIA children and their course in the circumstances of applied immunosuppressive therapy. Out of 51 patients with JIA, 10 (19.6%) patients had a SARS-CoV-2 infection which was most common in patients with sJIA, but with a similar course to the general pediatric segment, despite immunosuppressive therapy. In 3 out of 10 children, the infection exacerbated JIA, and an increase in therapy was imperative. Children with JIA and COVID-19 should be monitored to identify eventual long-run exacerbations after infection [157].

Since children and teens have not experienced very often and generally only mild forms of SARS-CoV-2 infection compared to adults, Sengler et al. aimed to study the gravity, the clinical features and the course of COVID-19 in pediatric patients under diverse therapies for their primary rheumatic and musculoskeletal disease (RMD), using a survey within the German national pediatric rheumatology database. Due to corrective suppression of the immune responses, these subjects may be endangered of an extreme evolution of SARS-CoV-2 infection, or a recurrence or intensification of the rheumatic disease triggered by COVID-19. Seventy-six patients with RMD and positive for SARS-CoV-2 infection (mean age 14 years), of whom only fifty-eight showed symptoms, including three patients with sJIA, all symptomatic, had a mild course of disease with good progression, i.e., in the vast majority (84%) no significant increase in RMD activity was observed, regardless of whether the immunosuppressive medication was kept or discontinued. Only two patients were hospitalized, one of whom required intensive care and died of cardio-respiratory failure. The authors concluded that COVID-19 does not appear to have a substantial impact on RMD activity in children, but the clinical features, severity and outcome in patients with RMD under various therapies are not yet easy to understand [158].

Finding a cure for COVID-19 was the most important life-saving task right from the start, and several vaccines have been developed, but none are 100% effective [159].

Parums D.V. analyzed the evolution of the pandemic and the new mutations that have emerged, which have shown that both vaccinated and unvaccinated, especially people with a compromised immune system, will continue being infected with SARS-CoV-2. For this reason, it has been a matter of the utmost importance for research to advance therapies that minimize the gravity of the infection in those who suffer and need hospitalization. Because IL-6 is a proinflammatory cytokine whose serum concentration is high in COVID-19, a humanized monoclonal antibody to the IL-6 receptor (IL-6R), TCZ, was granted an emergency authorization in June 2021 by the FDA, to treat inpatients with moderate and severe COVID-19, based on the results of two clinical trials, REMAP-CAP (NCT02735707) and RECOVERY (NCT04381936). TCZ has been authorized by the FDA since 2011 for the treatment of rheumatoid arthritis, JIA and other autoimmune and autoinflammatory diseases. The use of anti-SARS-CoV-2 monoclonal antibodies is now in clinical progress, being tested in clinical trials worldwide, even as combined antibodies to treat moderate and severe infections. New reliability and output data on IL-6 and IL-6R targeting will emerge to implement more individualized therapies that work better to control the systemic impact of COVID-19 [160].

Moreover, the European Medicines Agency (EMA) has recently approved Actemra/Roactemra (tocilizumab) and recommended it against severe forms of COVID-19. This drug is also an option to combat cytokine storms or exaggerated immune reactions induced by certain cellular treatments [161].

Because IL-6 plays a critical role in protecting the host from infections and tissue damage, and could serve as a valuable bioindicator in several specific cytokine storms, Kang et al. reviewed the present perspective on the multifarious roles of IL-6, receptors, and signaling pathways during severe inflammation which affects the whole body. IL-6 triggers the generation of various cytokines and chemokines by vascular endothelial cells, setting in motion the coagulation cascade, i.e., the endothelial cell disorder, featured by atypical coagulation and vascular leakage—an ordinary aggravating reaction in cytokine storms. Redundant IL-6 generation would conduct to chronic inflammatory diseases and hyperinflammation, such as cytokine storms. The authors point out that it has become increasingly clear that TCZ can successfully fulfill its mission of stopping IL-6 signaling with beneficial effects, not only in the management of cytokine storms in sJIA but also in other autoimmune and autoinflammatory disorders, including in sepsis and severe SARS-CoV-2 infection. Thus, IL-6, as a relevant pointer to cytokine storms, has been shown to control a variety of distinctive features linked to vascular homeostasis and inflammation. Blocking IL-6 signaling could be a helpful clinical master plan for different inflammatory conditions and induced cytokine storms. Regarding the interpretation of COVID-19 pathological studies, the use of TCZ has been established to be favorable in critically ill patients [162].

In the COVID-19 pandemic, other interleukin inhibitors, for example, anakinra—a recombinant humanized IL-1 receptor antagonist—may also ameliorate the effects of hyperinflammation, cytokine-associated storms (characterized by a life-threatening, fulminant hypercytokinaemia with high mortality), or lately, identified MIS-C in pediatric patients [163,164].

Phadke et al. investigated the intravenous administration of anakinra in the management of MAS secondary to sJIA, SLE or secondary hemophagocytic lymphohistiocytosis (sec-HLH) and others in 19 patients with a mean age of 13 years, indicating a possible positive effect in MAS/HLH critical cases and silencing of COVID-19-associated CSS or related MIS-C [165].

## 6. Discussions

Systemic juvenile idiopathic arthritis is a serious multifactorial autoinflammatory disease, characterized by arthritis or arthralgia lasting 2 weeks or longer (in the absence of arthritis). It is associated with extra-articular manifestations (high spiking fever of unknown origin, evanescent erythematous rash, generalized adenomegaly and/or splenomegaly and/or hepatomegaly, and serositis) which affects a percentage of 10–20% among children diagnosed with juvenile arthritis, with an unbalanced high rate of mortality through the appearance of MAS.

Treatment with high-dose steroids in short-term methylprednisolone boluses has been shown to be beneficial in controlling the disease, but in the long run, this therapy can cause serious side effects for patients. There are insufficient available prospective randomized studies in various JIA subtypes, including the most favorable management (i.e., dosage/delivery/length of therapy, and the process of gradually lessening) strategies [166,167].

Recent research has deepened the knowledge of pathophysiological mechanisms of sJIA, which has allowed the introduction of new and targeted treatments, namely bDMARDs, which have significantly changed the evolution and prognosis of this disease. New biotechnologically imagined bDMARDs are genetically modified to act like natural proteins in the child’s immune system, and their early administration would have the advantage of reducing the need for corticosteroids at the onset of sJIA. However, there are still many questions about the use or avoidance of glucocorticoids in relapse or complications, such as MAS, infections, long-term toxicity, and even a newly associated lung disease.

The COVID-19 pandemic has raised an issue, and it is still unclear whether patients with autoimmune inflammation from rheumatic diseases are at a higher risk of becoming infected with SARS-CoV-2 and whether they will have an unfavorable clinical course under bDMARDs therapy [168]. It was initially found that children are less likely to be affected by SARS-CoV-2 infection. However, after infection with this virus, at approximately 2–4 weeks, some cases of MIS-C have been reported. There are few data on the risk of a new coronavirus-induced JIA flare, however, there have been patients with inactive JIA or who are in remission, who have developed a sudden outbreak of their primary rheumatic disease in close temporal correlation with a confirmed COVID-19 diagnosis [169,170].

In an attempt to elucidate the as yet unknown effect of SARS-CoV-2 infection on the evolution of JIA, Hügle et al. studied 988 JIA cases from the database of the German Center for Pediatric and Adolescent Rheumatology from July 2020 to June 2021 and concluded that only 5 patients in remission or inactive disease had a flare-up of JIA four weeks after infection with SARS-CoV-2. A change in cytokines induced by SARS-CoV-2 infection could trigger a flare-up of JIA in a previously more susceptible person. The authors drew attention to the importance of raising awareness among pediatric rheumatologists to advise their patients of the dangers of infection with this virus and the role of vaccination against SARS-CoV-2 [171].

Angiotensin-converting enzyme 2 (ACE-2) is downregulated by the SARS-CoV-2 spike protein when the virus enters human endothelial cells. Afterwards, the resulted ACE reverses the identical underlying substrate into angiotensin II, a proinflammatory mediator. It generates a hyperinflammatory state, vasoconstriction and greater lung vascular permeability, which is a feasible cause of SARS-CoV-2 virus-induced pulmonary tissue injuries. Following the activation of T and B lymphocytes, an inflammatory process will be initiated with the release of proinflammatory factors (TNF-α, IL-1β, IL-2, IL-6, IL-10) and chemokines (MCP-1/CCL2, MIP -1A), which will recruit many monocytes/macrophages and will eventually result in a cytokine storm-like process [172,173].

ACE-2 diminishes with age [174] and, therefore, children and adolescents may have higher expression of ACE-2 with an anti-inflammatory effect, so they may be more prone to infection, but will generally develop asymptomatic forms. Thus far, until the Omicron variant, it was expected that children would most likely be asymptomatic in the case of SARS-CoV-2 infection; however, there is still a strong need for more data on the course of the disease in children with RMDs.

In a recently published study involving 180 subjects, Haşlak et al. studied the seroprevalence in asymptomatic SARS-CoV-2 children and adolescents, with childhood-onset rheumatic diseases, such as FMF, vasculitis, JIA and juvenile SLE, and compared it to healthy subjects, and found no significant difference with respect to the asymptomatic SARS-CoV-2 seropositivity rates between the investigated groups. In this study, 5 of 19 JIA patients were seropositive, but in spite of the fact that DMARD medication was not discontinued, all were completely asymptomatic. Thus, for the time of the pandemic, consideration of stopping the DMARDs, mainly the biologics, because of their suppressive effects on the immune system, is still in discussion. Correctly, each bDMARDs category should be assessed independently because of its own unique mechanisms of operation. Authors concluded that RMDs children and adolescents, even if they get immunosuppressive drugs, might remain asymptomatic during COVID-19, just like their healthy colleagues [175].

From the data analyzed so far, there have been no exacerbations of both COVID-19 and sJIA under bDMARDs treatment, but multicenter international studies are still needed to highlight all risk factors for a possible severe course of COVID-19 among sJIA patients. Children with sJIA and COVID-19 should be monitored to identify eventual long-run exacerbations after infection.

Indications for the therapy of patients with RMDs in the context of the COVID-19 pandemic issued so far unanimously by international associations are as follows: glucocorticoids can be used in minimal doses during COVID-19 infection; bDMARDs should be continued, but the treatment should be stopped only during infections, as per standard recommendations. Long-term protection for these patients is provided only by the vaccine [176].

COVID-19 does not appear to have a substantial impact on RMD activity in children, but the clinical features, severity and outcome in patients with RMDs under various therapies are not yet easy to understand, as Sengler et al. pointed out [158].

In the last ten years, special attention has been paid to the study of the “cytokine storm”, which has grown exponentially in the COVID-19 era, when it was found that a certain category of patients infected with SARS-CoV-2 showed cytokine storm-like manifestations, with life-threatening hyperinflammation and multi-organ dysfunction. MAS, which occurs in the circumstances of rheumatic pathologies, especially sJIA, is a model of CSS, whose management is effective when using steroids and biological anti-cytokine therapy (for example, TCZ, an anti-IL-6 biologic (a pillar of CSS treatment)), both in MAS and various forms of CSS. Although obvious clinical-paraclinical differences have been observed, especially with the occurrence of coagulopathy with vascular endothelial damage in COVID-19, the medical elite has used treatment prototypes from MAS and other CSS to develop the best management methods for these severely affected patients. While initially, the results of treatment with biological anti-cytokines (in uncontrolled case series of anti-IL-6 and anti-IL-1 biologics) in COVID-19 were encouraging, current, increasingly documented studies have shown more intricate pathogenic mechanisms and heterogeneous results. To better examine the pathophysiology of CSS, in a recent review, Ombrello et al. compared and discussed clinical symptoms, immunological data and drugs results that were applied for hyperinflammation triggered by SARS-CoV-2 infection, and MAS [133].

Because fulminant immunological disturbances and cytokine storms develop only in a certain category of patients with severe COVID-19, it is useful to implement clinical-biological models for identification and sorting which were formerly used in HLH and MAS. Previous experience in the management of CSS and MAS, respectively, allows a more effective approach in the therapy of inflammation and the relief of symptoms in patients with severe evolutionary COVID-19.

Applying high doses of anakinra has been shown to be safe and efficacious for patients with MAS when given immediately after starting corticosteroids [164,177].

Anti-IFN-γ therapy is already approved by the FDA for HLH using emapalumab, a monoclonal antibody that is being studied clinically and is also recommended for MAS in children with sJIA [178].

In a study of 131 patients hospitalized with moderate-to-severe COVID-19 pneumonia requiring oxygen support but not admitted to the intensive care unit, and who were randomly assigned to a TCZ group and usual care alone group (UC group), Hermine et al. proved an eventual drop in non-invasive ventilation, intubation or death at 14 days, but no disparity in the death rate at 28 days [179].

The COVID era has shown that there are serious cases in which the patient’s antiviral over-regulated response becomes harmful by itself, as in MAS. Even if the exact phenomena that produce and control the CSS associated with COVID-19 are still undefined, increased systemic inflammation is associated with worse outcomes in COVID-19, and that could be helpful for considering the patient’s prognosis.

This review highlights some of the ideas adopted by many other researchers around the world at which we subscribe that, as COVID-19 continues to manifest itself worldwide through the various mutations of the SARS-CoV-2 virus still present, better-developed guidelines for sJIA-MAS would elucidate fundamental and unanswered issues to date: why, what, how, when?

Why is life worth saving and which biomarkers predict the onset of MAS in sJIA or the onset of a severe COVID-19 cytokine storm?

What are the most important contributing factors?

Starting from CSS, how do we decide on the most appropriate therapeutic protocols and when should steroids and bDMARDs be administered?

Multicenter randomized controlled trials are still acutely necessary to establish at what time and by what means should immunoregulator products be administered in patients with a negative response to corticosteroids or who have contraindications [133].

McGonagle et al. mapped MAS in the COVID era and pointed out that, as a hyperinflammatory condition, MAS reaches its highest point through multiple interactive networks of infectious, genetic, immune deregulated, and innate immune defense factors. These could generate distinct conditions, such as sepsis, connective tissue disease, sJIA, AOSD and cancer [180]. Applying a continuous approach to an immunological disorders description that uses the barriers of innate and adaptive immunity, one can consider at one end, MAS with extinction or a “loss of immune function”, as in “perforinopathies” and in several sJIA-AOSD archetypes; and at the other end, hyperresponsiveness with a “gain of immune function” as in sJIA-AOSD being associated with the MHC class II and CAR T cell therapies (HLH/MAS) [180,181], which could also precipitate MAS.

This model establishes important criteria for evaluating severe COVID-19 pneumonia cases in which type I IFN immunity is disabled, and which favors the emergence of a second shock wave cytokine alveolitis with accompanying immunothrombosis (a two-way process that links coagulation and inflammation) [182]. This phenomenon is different from MAS, but with characteristics of “loss of immune function” suggested by the authors. This approach to MAS, which is explained as the “loss or gain of immune function”, paves the way for a new classification of MAS with important therapeutic implications [180].

Laboratory measurements in the 21st century in immunology are advanced and could determine cytokines of the order of femtograms per milliliter. Surprisingly, a quantitative definition for CSS has not yet been issued, i.e., a very high concentration of cytokines or hypercytokinemia, but customized only in certain specific clinical circumstances [183,184,185]. Even though the CSS is not defined exactly quantitatively, it can undoubtedly develop very quickly occasionally in different life-threatening pathologies, such as MAS.

It turned out that MAS is in a complex relationship with infectious diseases, insufficiently well defined, and in the case of COVID-19, MAS manifests itself as a distinct phenotype with hyperferritinemia and disseminated intravascular coagulation, for which we do not yet have a complete description [186].

At present, the treatments applied in MAS did not turn out to be beneficial in all severe COVID-19 cases and are currently insufficiently explained. Authors consider that the failures in the MAS treatment of severe COVID-19 patients are due to an incorrect understanding of the two different MAS immunopathologies—one with “loss” and the other with “gain” of immune function. Multiple viral infections, including SARS-CoV-2, can trigger MAS with diffuse hemorrhagic manifestations, which however differ from other CSS and MAS with consumptive coagulopathy. The newest anti-cytokine therapies targeted to sJIA-AOSD could soon stop this terrible MAS complication [180].

In both MAS phenotypes, T lymphocytes are involved. A distinction of the immune response should be done correctly in both cases, as a defective release of IFN-γ and other essential cytokines, such as IL-1, IL-6, IL-18, etc., for appropriate therapeutic conduct and prognosis.

MAS with hypercytokinemia involves the activation of macrophages in the sinusoids of the bone marrow, liver and spleen, by direct phagocytosis of hematopoietic cells. Identifying other versions of MAS by considering the perivascular anatomical arrangement of activated macrophages in the above-mentioned structures could establish other conditions with hypercytokinemia. Moreover, sJIA and AOSD are considered autoinflammatory diseases, but may also turn into “autoimmune inflammation” [41,187]. Given the role of the lymphoid cells, MAS, in conjunction with these two pathologies, is totally different from the entity that occurs in severe cases of COVID-19 [180].

Research on MAS and COVID-CS has found substantially different clinical features and laboratory parameters, such as hyperferritinemia and serum CRP levels in severe cases of COVID-19 being lower than in MAS, while the levels of the most important cytokines IL -1, IL-6, IL-18, etc., have lung-specific immune characteristics, different from a typical CSS [141,188,189].

Another important difference is that MAS is accompanied by DIC, but the macrophages activated in COVID-19 act specifically on the lung, inducing alveolitis with immunothrombosis, called pulmonary intravascular coagulopathy (PIC), which is different from DIC [189].

Dysregulated immune reactions will generate a massive agglomeration of immune cells and signaling factors that will trigger a huge release of cytokines that will flood the lung space especially, which differs from a systemic cytokine storm [190].

Former clinical trials on flu with a fatal course showed 1000-fold higher levels of IL-1β and IL-6 in broncho-alveolar lavage fluid than in blood, whereas IFN-γ levels were just a bit higher, but of the same value both in lung and serum. These aspects suggest the existence of lung-distinctive intrinsic cytokine, which is set free in motion to trigger the disease, unlike the MAS phenotypes in sJIA and AOSD [191].

McGonagle et al. highlighted that in severe COVID-19 infection, there is a low concentration of IFN-γ and its barricade could be explained by the overlapping characteristics of florid MAS, including juxtaposed hyperferritinemia. Attacking other important cytokines in MAS may also be unproductive due to infection with severe lung damage and multiple versatile reactions with lung cell-specific cytokines. MAS and hypercytokinemia may be triggered by the antigen-presenting dendritic cells and T cells in a usual MAS. However, in severe COVID-19, the first-wave type-I IFN reactions are not able to control the SARS-CoV-2 infection, and a subsequent wave of strong proinflammatory cytokines is generated due to powerful myeloid infiltration, followed by potent local immune responses inside pulmonary cells and else. In this second surge, IFN-γ is not especially raised, but numerous myeloid-connected and tissue-generated cytokines are increased. In contradistinction to classical MAS, the concentrations of ferritin and cytokines are not extremely high, but in certain situations of the infectious process, severe MAS could occur and evolve. Therapy for MAS has been applied based on facts and observations by practical experience in medical science. At present, the biologics acting anti-IL-6 are efficient only for MAS, which occurs during immunotherapy in cancers, but not for MAS related to sJIA and AOSD.

In experimental studies on MAS in oncology, sJIA and AOSD, it has been observed that blocking the IL-1 receptor (IL-1Ra) has a beneficial effect; however, stopping IL-1β is beneficial for sJIA, but not for MAS that may occur in sJIA. Similarly, when an IFN-γ antagonist was administered in MAS from sJIA and pHLH, satisfactory results were obtained, but it cannot be argued that this treatment regimen can be successfully applied to viral lung infection. In MAS associated with sJIA-AOSD, short term steroid therapy has an inhibitory effect on a wide range of cytokines and on JAK-STAT pathways and therefore could have a major impact on systemic inflammation [180,192,193].

For example, the RECOVERY Collaborative Group demonstrated in a clinical trial, with 2104 patients designated to receive dexamethasone and 4321 to receive conventional care, that steroid therapy was successful in reducing the 28-day mortality, but only in severe cases who needed invasive mechanical ventilation or only oxygen, but not in patients who did not need respiratory assistance [194].

The difference between MAS and MIS-C is due to the presence in the first case of hyperferritinemia, anemia and coagulopathy, features that are missing in MIS-C, but which are accompanied by a large number of proinflammatory factors and cardiogenic shock with reduced ejection fraction [32].

Yener et al. comparatively studied the clinical-biological parameters and evolution in 244 patients with MIS-C, KD and sJIA-MAS, for establishing an early diagnosis and correct treatment. From a clinical point of view, the cardiovascular, gastrointestinal, neurological and muscle-pain symptoms were more severe in MIS-C than in KD, or sJIA-MAS. At the same time, children with MIS-C had pronounced lymphopenia, with elevated acute phase reactants (CRP, ESR and NT-pro-brain natriuretic peptides or pro-BNP). Mean serum ferritin levels were very high in patients with sJIA-MAS (10,442 ng/mL), lower in MIS-C (440 ng/mL) and lowest in KD (170 ng/mL) [195].

Compared to MAS, ferritin levels in MIS-C are lower and fibrinogen is higher. In sJIA, high levels of ferritin and low levels of fibrinogen are inceptive evidence of an inevitable sJIA-MAS [196].

In this pandemic, substantial efforts have been made around the world using sequencing technologies to discover the genomic variants involved in the interindividual multitude of clinical manifestations of COVID-19 phenotypes, especially severe forms. Recently, for increased reliability and precision, researchers have developed optical genome mapping techniques for patients with COVID-19 in order to identify the genes involved in host-viral interaction mechanisms, innate immunity, inflammatory response, viral replication and severity of the disease [197].

A hope for patients with JIA who do not respond to biologics, or to potentiate it for better results, is photobiomodulation therapy, which when creatively applied, opens new avenues for quantified regulation of the immune system [198,199,200], and thus, sometimes avoiding the emergent adverse reactions to bDMARDs, for example, sJIA-LD, a novel parenchymal lung disease, increasingly detected in sJIA [98,99].

**Figure 1:**
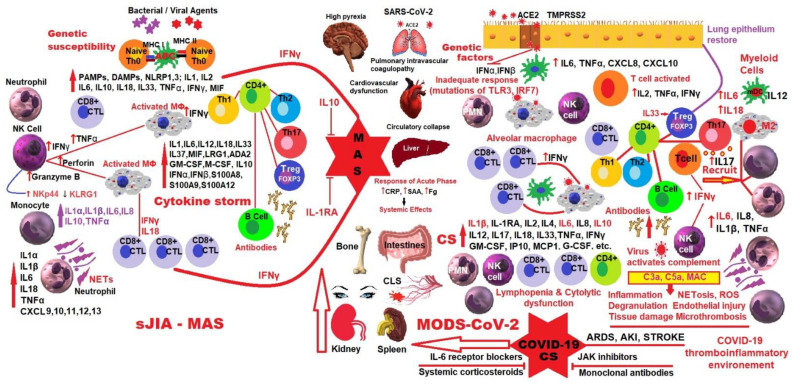
Complex cytokine storms in sJIA-MAS and COVID-19-CS and secondary organ dysfunction, ↑ Increased; ↓ Decreased (the figure was imagined and drawn by L.M.A. using Microsoft Paint 3D (3D Library—Biology: human heart and brain) for Windows 10 and also using completely free picture material (human lungs, kidney, eyes, intestines, CLS and bone clip arts) from SeekPNG.com (accessed on 31 January 2022), for which we are very grateful).

The comparative representation of the cytokine storm picture in sJIA-MAS and COVID-19 CS with the multiple organ dysfunctions in both pathologies are highlighted in (Figure 1).

## 7. Conclusions

Over the past two years, researchers around the world have been studying intensively how to cope with the new SARS-CoV-2 virus and the fulminant diseases triggered by infection with this virus.

The COVID era has demonstrated that there are serious cases in which the patient’s antiviral over-regulated response becomes harmful by itself, as in sJIA-MAS.

Even if the exact phenomena that produce and control the CSS associated with COVID-19 are still undefined, increased systemic inflammation is associated with worse outcomes in COVID-19, and that could be helpful for considering the patient’s prognosis.

At present, current treatments have not been shown to be fully effective in all severe cases of COVID-19 and MAS. This review draws attention to the fact that the failures in the treatment of MAS in sJIA patients, and those with severe COVID-19 could be due to a misunderstanding of the two different and opposed MAS immunopathologies—one with a loss of immune function, and the other with a gain of immune function.

MAS, in conjunction with these two pathologies, is totally different from the entity that occurs in severe cases of COVID-19, which is a unique immunological process related to the lungs.

It is considered that MAS reaches its highest point through multiple interactive networks of infectious, genetic, and innate immune deregulated factors that could generate distinct conditions, such as sepsis, sJIA, AOSD, cancer, etc.

It has turned out that MAS is in a complex relationship with infectious diseases and is insufficiently well defined, and in the case of COVID-19, MAS manifests itself as a distinct phenotype, for which we do not yet have a complete description.

As there is still a risk of new SARS-CoV-2 virus mutations, measures are needed to educate families, children, teachers and civil society to better cope with the impact of COVID-19 and the new challenges.

Researchers, medical doctors and especially pediatric rheumatologists need to develop new integrative diagnostic and treatment approaches to further support children with RMDs and COVID-19.

Psychological, educational and especially financial support is required for children who have lost one, both parents, grandparents and legal caregivers in this pandemic.

Global action is needed to overcome inequities in health care, even from an early age for children in the poorest countries, to positively influence present and future development, to generate hope and not fear.

In the future, research is needed for children and adolescents with RMDs in order to optimize their health and safety in the COVID era.

Multicenter randomized controlled trials are still acutely necessary to establish at what time and by what means should immunoregulator products be administered in sJIA-MAS patients with a negative response to corticosteroids or contraindications.

## Figures and Tables

**Table 1:** Clinical symptoms, biomarkers and targets for sJIA.

Cardinal Symptoms	Other Systemic Manifestations	Biological Parameters	Life-Threatening Complications for Patients
Fever for at least 2 weeks	Mucous lesions (odynophagia)	↑ ESR and ↑ CRP levels	MAS (10% apparent, 40% subclinical)
Skin evanescent rash (diffuse macular, scarlatiniform, urticarial)	Generalized lymph node enlargement	↑ Serum ferritin ↓ Glycosylate ferritin	Fulminant hepatitis
Leukocytes ≥ 10,000 mm^3^ Neutrophils ≥ 80%	Gastrointestinal symptoms (abdominal pain, hepato-splenomegaly)	↑ D-dimer	Disseminated intravascular coagulation
Arthritis in one or more joints or Arthralgia	Heart involvement (pericarditis, myoca2-glycoproteinrditis, coronary aneurysm)	↓ Fg	Thrombotic microangiopathy
	Lung damage (pneumonia, pleurisy)	↑ LDH, ↑ AST, ↑ ALT	Cardiac tamponade/myocarditis
	Myalgia/Myositis	Coagulation disorders	Acute respiratory failure syndrome
	Hepatitis		
	Uveitis (is quite rare)		

C-reactive protein (CRP); erythrocyte sedimentation rate (ESR); fibrinogen (Fg); lactic acid dehydrogenase (LDH); aspartate aminotransferase (AST); alanine aminotransferase (ALT). ↑ Increased; ↓ Decreased.

**Table 2:** Positive and differential diagnosis for sJIA. Fever of unknown origin prolonged, daily, in bursts of ≥39 °C for a period of at least two weeks plus two major criteria; or one major criterion and two minor criteria.

**Major criteria**	Evanescent rash Arthritis			
**Minor criteria**	Generalized adenomegaly and/or splenomegaly and/or hepatomegaly	Arthralgia	Serositis (pleural, pericardial or peritoneal)	Leukocytosis (≥15,000/mm^3^) with neutrophilia
**Differential diagnosis for sJIA subtypes**	(a) Psoriasis or a history of psoriasis in the patient or in a first-degree relative. (b) Arthritis in an HLA-B27-positive boy beginning after the sixth birthday. (c) Ankylosing spondylitis, enthesitis-related arthritis (ERA), sacroiliitis with inflammatory bowel disease or acute anterior uveitis, or a history of one of these disorders in a first-degree relative. (d) Association with 2 positive tests for immunoglobulin M rheumatoid factor (IgM-RF) at least 3 months apart.			
**Other entities excluded for positive diagnosis**	**Infections**	**Malignancy and autoimmune disease**	**Vasculitis**	**Autoinflammatory diseases with periodic familial fevers**
	Generalized infections: septicemia; Deep infections: osteomyelitis or abscesses; Causes of prolonged fever: endocarditis, yersiniosis, tuberculosis, brucellosis; Post-infectious arthritis: Group A beta-hemolytic Streptococci (GAS) Viruses: Epstein-Barr virus (EBV), Cytomegalovirus (CMV), Human Immunodeficiency Virus (HIV) parvovirus B19, hepatitis, measles; human herpesvirus 8 associated multicentric Castleman disease (HHV-8-associated MCD).	Leukemia, lymphoma, neuroblastoma etc. Polyarticular JIA with systemic manifestations or Systemic Lupus Erythematosus (SLE), juvenile dermatomyositis.	Polyarteritis nodosa, KD.	Mediterranean fever (FMF) caused by mutations of MEFV gene; tumor necrosis factor receptor-associated periodic fever syndrome (TRAPS), mevalonate kinase deficiency (MKD) by mutations of the mevalonate kinase gene; cryopyrin-associated periodic syndromes (CAPS), by mutations of NLRP3; periodic fever, aphthosis, pharyngitis and adenitis (PFAPA).

**Table 3:** Criteria for classification of MAS in patients with sJIA at the initiative of EULAR/ACR/PRINTO collaborative organizations.

High, Prolonged Fever of Known Origin in a Known or Suspected sJIA Patient may be Considered an Essential Element in the Diagnosis of MAS if Associated with the Following Criteria:	
Ferritin > 684 ng/mL and Any Two of the Following Criteria:	
	Platelet count ≤ 181 × 10^9^/L
	Aspartate aminotransferase (AST) > 48 unites/L
	Triglycerides > 156 ng/dL
	Fibrinogen ≤ 360 ng/dL

## Data Availability

Not applicable.

## Abbreviations

AKI	Acute kidney injury
ARDS	Acute respiratory distress syndrome
ADA2	Adenosine deaminase 2
AOSD	Adult-onset Still’s disease
AGEs	Advanced glycation end products
AHSP	Alpha hemoglobin–stabilizing protein
ACR	American College of Rheumatology
ACE-2	Angiotensin-converting enzyme 2
ANKRD55	Ankyrin Repeat Domain 55
APC	Antigen-presenting cell
ANA	Antinuclear antibodies
AST	Aspartate aminotransferase
AID	Autoinflammatory disease
BCL6	B-cell lymphoma 6 protein, encoded by the BCL6 gene
bDMARDs	Biologic disease-modifying antirheumatic drugs
CP	Calprotectin
CLS	Capillary leak syndrome
CASP8	Caspase-8
CCL23	C-C Motif Chemokine Ligand 23
CDC	Centers for Disease Control and Prevention
CXCL1	Chemokine (C-X-C motif) ligand 1
CCL2	Chemokine (C-C motif) ligand 2
CID	Clinically inactive disease
CD6	Cluster of Differentiation 6
CD163	Cluster of Differentiation 163
C3a	Complement component 3a
C3b	Complement component 3b
cNK	Conventional NK cells
CNVs	Copy number variations
CRP	C-reactive protein
COVID-CS	Cytokine storm COVID-19
CSS	Cytokine storm syndrome
CMV	Cytomegalovirus
CTL	Cytotoxic T-Lymphocyte
CTLA4	Cytotoxic T-Lymphocyte Associated Protein 4
DAMP	Damage-associated molecular pattern
DAMPs	Danger (or damage)-associated molecular patterns
DMARDs	Disease-modifying antirheumatic drugs
DIC	Disseminated intravascular coagulation
ELP	Endogenous lipoid pneumonia
EHEC	Enterohemorrhagic Escherichia coli
ERA	Enthesitis-related arthritis
EBV	Epstein-Barr virus
EBV-HLH	Epstein-Barr virus-induced hemophagocytic lymphohistiocytosis
ESR	Erythrocyte sedimentation rate
ALAS2	Erythroid-specific 5-aminolevulinate synthase
EULAR	European League Against Rheumatism
FMF	Familial Mediterranean Fever
FasL	Fas ligand
Fg	Fibrinogen
FDA	Food and Drug Administration
GWAS	Genome-wide association study
GF	Glycosylated ferritin
G-CSF	Granulocyte colony stimulating factor
GM-CSF	Granulocyte-macrophage colony stimulating factor
GAS	Group A beta-hemolytic Streptococci
HSPB1	Heat shock protein beta-1
HSC	Hematopoietic stem cell
HLH	Hemophagocytic lymphohistiocytosis
HMGB1	High mobility group box-1
hsCRP	High-sensitivity C-reactive protein
HIV	Human Immunodeficiency Virus
HLA	Human leukocyte antigen
IgM-RF	Immunoglobulin M rheumatoid factor
H1N1	Influenza A virus subtype H1N1
ILCs	Innate lymphoid cells
ILC1s	Innate lymphoid cells group 1
IFN-γ	Interferon-gamma
IRF	Interferon regulatory factors
IL6ST	Interleukin 6 Signal Transducer
IL-18BP	IL-18 binding protein
IV	Intravenous
KD	Kawasaki disease
KDSS	KD shock syndrome
KIRs	Killer cell immunoglobulin-like receptors
KLRF1	Killer cell lectin-like receptor F1
KLRB1	Killer cell lectin-like receptor subfamily B member 1
KLRG1	Killer cell lectin-like receptor subfamily G member 1
KITLG	KIT ligand
KLF1	Kruppel-like factor 1
LDH	Lactic acid dehydrogenase
LRG	Leucine-rich *α*2-glycoprotein
LPS	Lipopolysaccharide
LD	Lung disease
TNFSF1	Lymphotoxin-alpha (LT-α) = tumor necrosis factor-beta (TNF-β)
MAS	Macrophage activation syndrome
sJIA-MAS	Macrophage activation syndrome in sJIA
MIF	Macrophage migration inhibitory factor
MHC	Major histocompatibility complex
MMP-1	Matrix metalloproteinase 1
MEFV	Mediterranean fever
MAC	Membrane attack complex
MTX	Methotrexate
MCP1	Monocyte chemoattractant protein 1
MODS-CoV-2	Multiple organ dysfunction in SARS-CoV-2
MIS-C	Multisystem inflammatory syndrome in children
RMD	Musculoskeletal rheumatic disease
MPO-DNA complexes	Myeloperoxidase-DNA complexes
mDC	Myeloid dendritic cell
MRP	Myeloid-related protein
NK	Natural killer
NKT cells	Natural killer T cells
cNK	Conventional NK
trNK	Tissue-resident NK
NETs/NETosis	Neutrophil extracellular traps
NLR	NOD-like receptor
nsJIA	Non-systemic forms of JIA
NOD	Nucleotide-binding oligomerization
NLRP	Nucleotide-binding oligomerization domain, Leucine rich Repeat and Pyrin domain
NLRP3	Nucleotide-binding oligomerization domain-like receptor pyrin domain-containing protein 3
NLRs	Nucleotide-binding oligomerization domain-like receptors = NOD-like receptors
OSM	Oncostatin M
PAMP	Pathogen-associated molecular pattern
PRRs	Pattern recognition receptors
PIMS-TS	Pediatric Multisystem Inflammatory Syndrome temporarily associated with SARS-CoV-2
PRINTO	Pediatric Rheumatology International Trials Organization
PRF1	Perforin 1
PBMC	Peripheral blood mononuclear cell
PMN, PML, or PMNL	Polymorphonuclear leukocytes
pHLH	Primary Hemophagocytic Lymphohistiocytosis
PCT	Procalcitonin
PKC	Protein kinase C
PAP	Pulmonary alveolar proteinosis
PIC	Pulmonary intravascular coagulopathy
RAGE	Receptor for advanced glycation end products
rIL-1Ra	Recombinant IL-1 receptor antagonist
rr	Reference range
RMD	Rheumatic and musculoskeletal disease
RUNX1	Runt-related transcription factor 1
RUNX3	Runt-related transcription factor 3
sec-HLH	Secondary Hemophagocytic Lymphohistiocytosis
SAA	Serum Amyloid A
STAT	Signal Transducer and Activator of Transcription
STAT4	Signal Transducer and Activator of Transcription 4
SNP	Single Nucleotide Polymorphism
SIRT2	Sirtuin 2
sJIA-LD	sJIA-associated lung disease
sRAGE	Soluble receptor for advanced glycation end products
SULT1A1	Sulfotransferase 1A1
SLE	Systemic lupus erythematosus
SURFS	Systemic undifferentiated recurring fever syndromes
TIM-3	T-cell immunoglobulin mucin-3
TMA	Thrombotic microangiopathy
trNK cells	Tissue-resident (tr)NK cells
TWEAK	TNF-like weak inducer of apoptosis
TCZ	Tocilizumab
TLR	Toll-like receptor
TGF-*β*	Transforming growth factor beta
TMPRSS2	Transmembrane serine protease 2
TAMs	Tumor-associated macrophages
TNF-α	Tumor necrosis factor alpha
TNFR1	Tumor necrosis factor receptor 1
TRAF1/C5	Tumor necrosis factor-receptor associated factor 1 and C5 (complement component 5)
TRAPS	Tumor necrosis factor receptor-associated periodic fever syndrome
PTPN2	Tyrosine-protein phosphatase non-receptor type 2
UNC13D	Unc-13 Homolog D
WGCNA	Weighted gene co-expression network analysis
**↑**	Increased
↓	Decreased
